# Supplementary material for: Targeted sortase A inhibition by novel peptidomimetic antivirulents against staphylococcal infections
Source: Microbiol Spectr. 2026 Mar 3;14(4):e02327-25. doi: 10.1128/spectrum.02327-25 (PMC13055291; doi:10.1128/spectrum.02327-25)
Supplement: Supplemental figures — Fig. S1 to S5. [file spectrum.02327-25-s0001.docx]

**Supporting Information**

**Targeted Sortase A Inhibition by Novel Peptidomimetic Antivirulents against Staphylococcal Infections**

Jordi C. J. Hintzen, Shadi Rahimi, Daniel Tietze, Jian Zhang, Priyanka Mehra, Ivan Mijakovic and Alesia A. Tietze

1. **Analytical peptide data:**

| Peptide | Calculated  Mw  [g/mol] | Experimental  Mw  [g/mol] | ppm | tR  [min] |
| --- | --- | --- | --- | --- |
| BzLPRDSar | 674.3626 | 674.3655 | 4.3 | 5.22 |


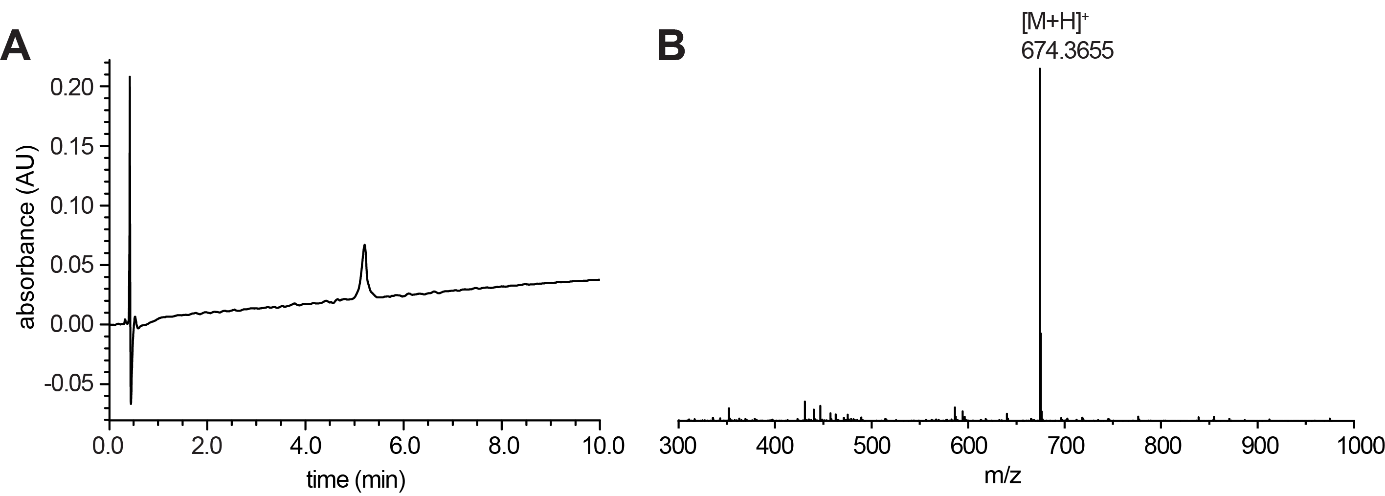


Fig. S1: A) HPLC chromatogram (gradient from 5 to 50% ACN in water over 10 min at 2 ml/min, detection at 214 nm) and B) high resolution mass spectrum for peptide 18 BzLPRDSar.

1. **Growth profiling *K. pneumoniae*:**

**Fig. S2:** Growth profiling curve of *K. pneumoniae* incubated in presence of varying concentrations of BzLPRDSar. Absorbance was measured at 600 nm as a measure of OD. All samples were measured in triplicate, error bars reported as standard error (±SE).

1. **Visual Representation of crystal violet staining.**


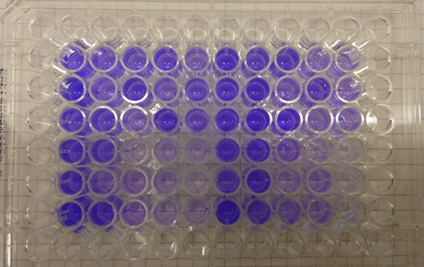


**Fig. S3:** Visual representation of a Crystal Violet staining assay to determine the extent of biofilm inhibition.

1. **Additional SEM images *S. aureus* and *S. epidermidis*:**


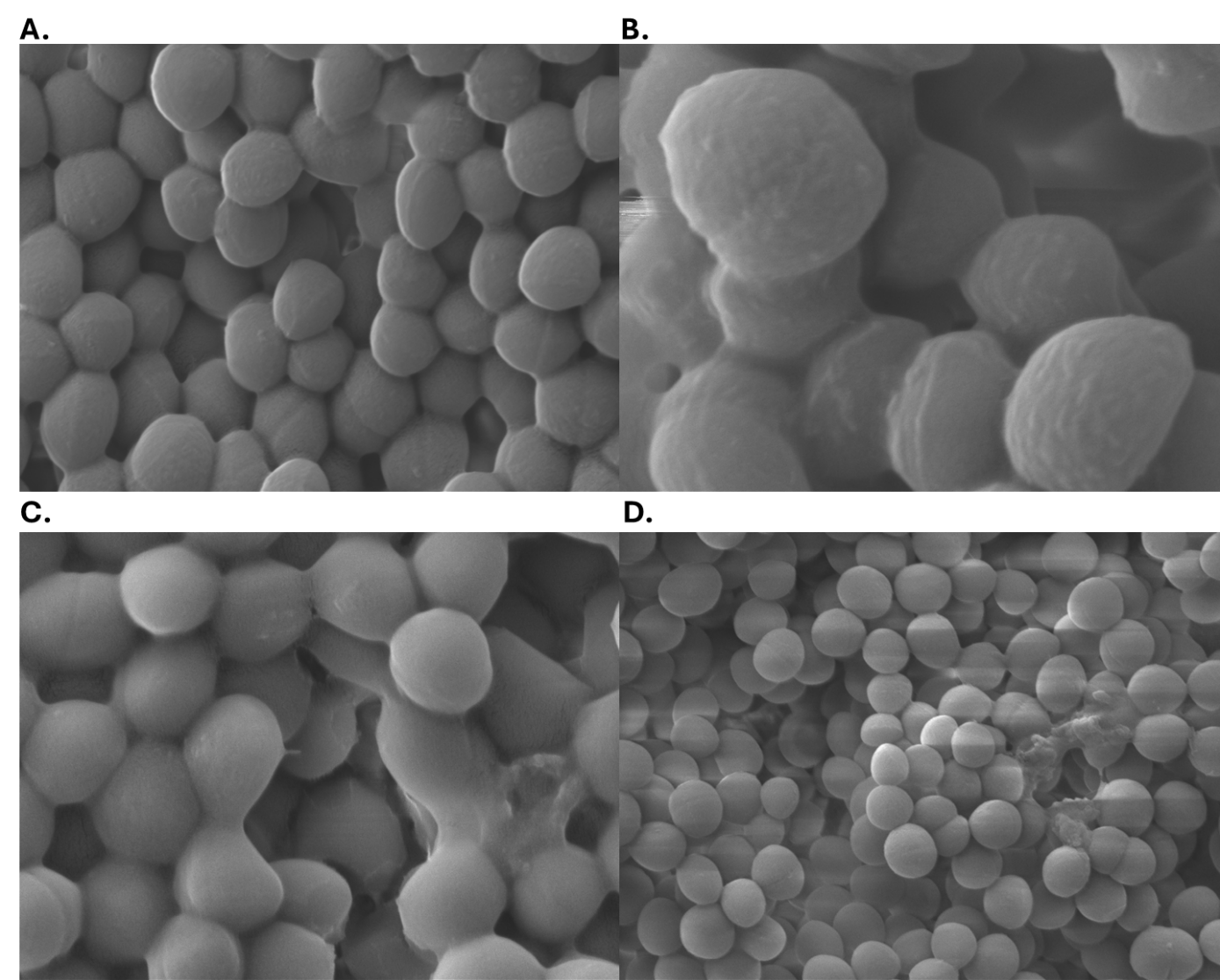


**Fig. S4:** SEM images illustrating structural changes in biofilms of *S. aureus* and *S. epidermidis.* **A)** *S. aureus* biofilms, untreated control, magnification 25,000x; **B)** *S. aureus* biofilms, incubated with 128 µg/ml of BzLPRDSar for 18 hours, magnification 50,000x; **C)** *S. epidermidis* biofilms, untreated control, magnification 25,000x; **B)** *S. epidermidis* biofilms, incubated with 128 µg/ml of BzLPRDSar for 18 hours, magnification 15,000x;


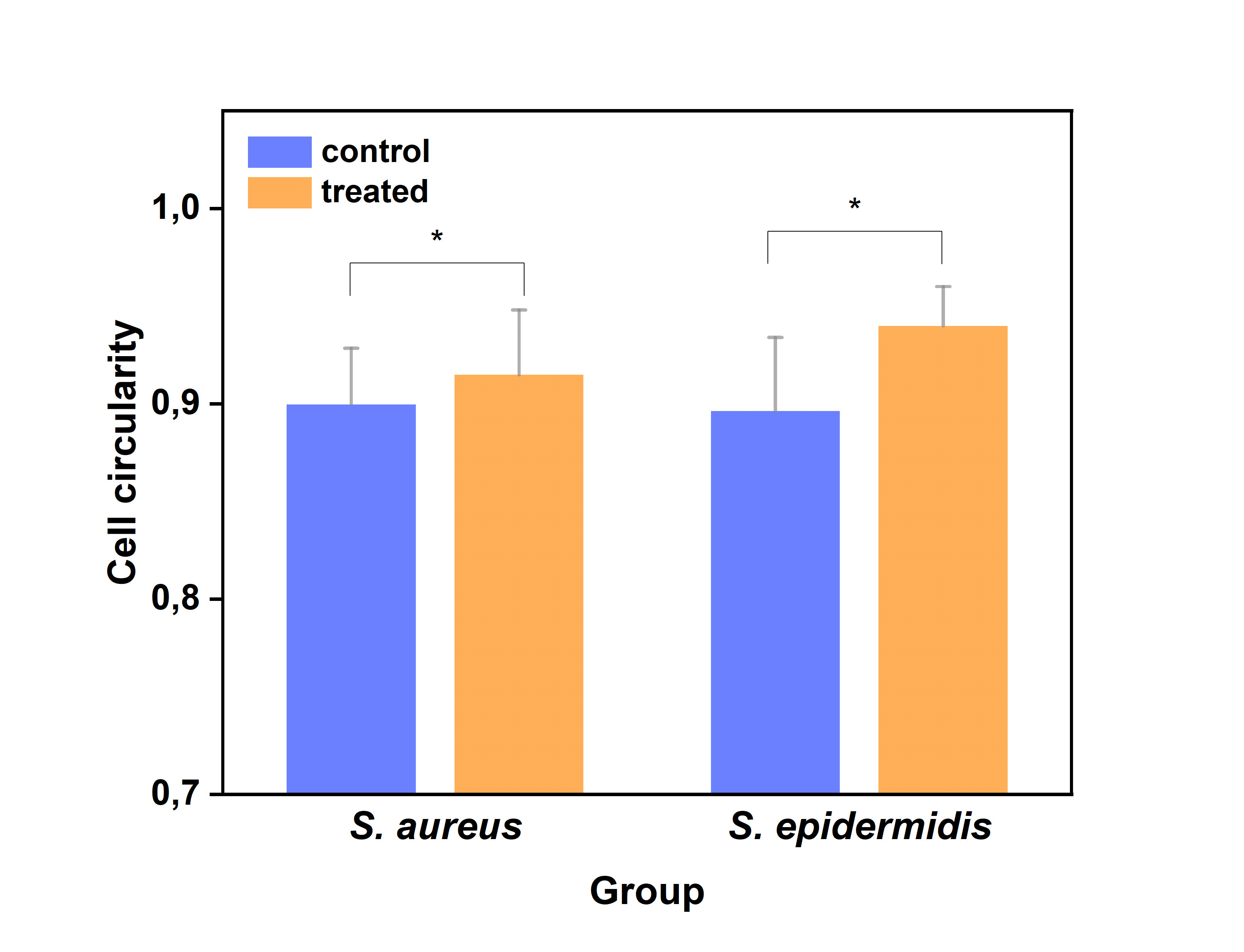


**Fig. S5:** Analysis of cell circularity after BzLPRDSar treatment at 128 μg/mL. Data are presented as as mean ± SD. A total of n = 50 cells per condition were analyzed. Statistical significance was assessed using the two-tailed Mann–Whitney U test.
